# Benzyl Cyanide Leads to Auxin-Like Effects Through the Action of Nitrilases in *Arabidopsis thaliana*

**DOI:** 10.3389/fpls.2018.01240

**Published:** 2018-08-24

**Authors:** János Urbancsok, Atle M. Bones, Ralph Kissen

**Affiliations:** Department of Biology, Norwegian University of Science and Technology, Trondheim, Norway

**Keywords:** *Arabidopsis thaliana*, auxin, benzyl cyanide, benzylglucosinolate, glucosinolates, myrosinase, nitrilase, phenylacetic acid

## Abstract

Plants within the Brassicales order generate glucosinolate hydrolysis products that can exert different biological effects on several organisms. Here, we evaluated the physiological effects of one of these compounds, benzyl cyanide (phenylacetonitrile), when exogenously applied on *Arabidopsis thaliana*. Treatment with benzyl cyanide led to a dose-dependent reduction of primary root length and total biomass. Further morphological changes like elongated hypocotyls, epinastic cotyledons, and increased formation of adventitious roots resembled a severe auxin-overproducer phenotype. The elevated auxin response was confirmed by histochemical staining and gene expression analysis of auxin-responsive genes. Nitriles are converted by specific enzymes, nitrilases (NIT1-3), to their corresponding carboxylic acids. The nitrilase mutants *nit1* and *nit2* tolerated benzyl cyanide treatments better than the wild type, with *nit2* being less resistant than *nit1*. A *NIT2*RNAi line suppressing several nitrilases was resistant to all tested benzyl cyanide concentrations. When exposed to phenylacetic acid (PAA) – the corresponding carboxylic acid of benzyl cyanide – wild type and mutant seedlings were, however, equally susceptible and showed a more severe auxin phenotype than upon cyanide treatment. Here, we demonstrate that the auxin-like effects triggered by benzyl cyanide on Arabidopsis are due to its nitrilase-mediated conversion to the natural auxin PAA.

## Introduction

Glucosinolates (GSLs) are nitrogen- and sulfur-containing secondary metabolites of plants belonging to the Brassicales order. GSLs are biologically inactive but many biological effects have been documented for their degradation products generated by the activity of β-thioglucosidases called myrosinases (Bones and Rossiter, 1996). As substrates and enzymes are spatially separated in intact tissue this hydrolysis usually happens when plant tissue is ruptured by mechanical damage or herbivory (Kissen et al., 2009). About 130 natural GSLs have been identified (Agerbirk and Olsen, 2012) and this complexity is exacerbated by the fact that their hydrolysis can result in one or more products such as isothiocyanates (ITCs), thiocyanates, epithionitriles, and nitriles (Bones and Rossiter, 2006).

Benzylglucosinolate, also known as glucotropaeolin, is a phenylalanine-derived GSL that can for example be found in nasturtium (*Tropaeolum majus*), garden cress (*Lepidium sativum*), white mustard (*Sinapis alba*), and papaya (*Carica papaya*; Cole, 1976; Lykkesfeldt and Møller, 1993; Bennett et al., 1997; Agerbirk et al., 2008). The hydrolysis of benzylglucosinolate can lead to benzyl-ITC, benzyl thiocyanate, or phenylacetonitrile (also called benzyl cyanide; Virtanen, 1965; Cole, 1976; Gil and Macleod, 1980a,b). Which of these products are formed depends on the plant species, the organ and the developmental stage of the plant, the presence of nitrile-specifier proteins (NSPs) or thiocyanate-forming protein (TFP), as well as cofactors and the reaction conditions (e.g., pH, iron ions; Burow et al., 2007; Wittstock and Burow, 2010; Kuchernig et al., 2012).

In addition to damage-induced hydrolysis of GSLs, their turnover in intact tissue has been postulated based on changes in GSL composition during the plant life cycle (Clossais-Besnard and Larher, 1991; Brown et al., 2003). A turnover of GSLs by myrosinases, generating nitriles that are further converted into carboxylic acids by nitrilases (NITs) was suggested (James and Rossiter, 1991; Bestwick et al., 1993). Nitrilases (EC 3.5.5.X) catalyze the hydrolytic cleavage of organic cyanides (nitriles) into carboxylic acids, with the release of ammonia (Pace and Brenner, 2001; Brenner, 2002). The first nitrilase was prepared from barley leaves (Thimann and Mahadevan, 1964; O’Reilly and Turner, 2003) and since then nitrilases have been identified in many plant species and microorganisms (Piotrowski, 2008). Arabidopsis possesses four nitrilase genes. *NIT1* (*At3g44310*), *NIT2* (*At3g44300*), and *NIT3* (*At3g44320*) encode enzymes with activity on GSL-derived nitriles when expressed *in vitro* (Bartling et al., 1992; Vorwerk et al., 2001; Osswald et al., 2002; Schreiner et al., 2010). *NIT4* (*At5g22300*) encodes an enzyme with a strong substrate specificity for β-cyano-L-alanine, an intermediate in cyanide detoxification (Piotrowski et al., 2001). NIT1–NIT3 homologs seem to be restricted to Brassicaceae whereas NIT4 homologs are found in all plant species (Janowitz et al., 2009).

Glucosinolate-derived nitriles are believed to be biologically less active than their corresponding ITCs (Wittstock et al., 2003). Production of nitriles instead of ITCs seems indeed to make plants more susceptible to generalist herbivores (Lambrix et al., 2001). But some nitriles have been shown to be attractive to insect pests and/or their parasitoids (Bartlet et al., 1997; Smart and Blight, 1997; Mumm et al., 2008; Pope et al., 2008; Kugimiya et al., 2010) and higher levels of indole-3-acetonitrile (IAN) deterred oviposition of the specialist *Pieris rapae* (de Vos et al., 2008). Also, benzyl cyanide (Bz-CN) was more toxic than the corresponding ITC when used as fumigant on the house fly (*Musca domestica*) and the lesser grain borer (*Rhyzopertha dominica*; Peterson et al., 1998).

The major biosynthetic pathway of auxin (indole-3-acetic acid or IAA) consists of the formation of indole-3-pyruvic acid (IPA) from tryptophan followed by the oxidative decarboxylation to IAA (Mashiguchi et al., 2011; Stepanova et al., 2011; Won et al., 2011). Other tryptophan-dependent biosynthetic pathways may contribute to IAA production (for review: Korasick et al., 2013; Kasahara, 2015). One of these pathways proceeds from tryptophan via indole-3-acetaldoxime to IAN which is converted to IAA by nitrilases (Zhao et al., 2002; Sugawara et al., 2009). In another pathway, tryptophan leads to the formation of indole-3-acetamide which is further converted to IAA (Pollmann et al., 2003, 2009). Tryptophan-independent pathways for the biosynthesis of IAA have also been proposed (for review: Korasick et al., 2013; Kasahara, 2015).

Phenylacetic acid (PAA) has long been known to exert an auxin-like effect on plants when applied exogenously (Haagen-Smit and Went, 1935; Zimmerman and Wilcoxon, 1935; Koepfli et al., 1938; Muir et al., 1967; Wheeler, 1977; Leuba and Letourneau, 1990). In addition, PAA is one of the few naturally occurring auxins and has been detected in several plant species, including Arabidopsis (Wightman and Lighty, 1982; Sauer et al., 2013; Sánchez-Parra et al., 2014; Sugawara et al., 2015). It was recently shown that PAA can be produced *in vitro* from phenyl pyruvate (PPA) by the activity of plant YUCCA (YUC) proteins and that the overexpression of *YUC*s led to increased PAA levels in Arabidopsis (Dai et al., 2013; Sugawara et al., 2015). Bz-CN was also shown to trigger auxin-like effects such as stimulating the elongation of oat coleoptiles, wheat coleoptiles, and *L. sativum* hypocotyls and stimulating adventitious root formation of *L. sativum* hypocotyls (Steiner, 1948; Wheeler, 1977). Although Steiner (1948) left open the possibility that Bz-CN was the active compound, he hypothesized that the hydrolysis of Bz-CN, probably inside the plant, generated the active PAA (Steiner, 1948). As mentioned before, recombinant nitrilases of the NIT1-NIT3 subgroup accept various aliphatic and aromatic nitriles, including Bz-CN, as substrate *in vitro* (Bartling et al., 1992; Vorwerk et al., 2001; Osswald et al., 2002; Ishikawa et al., 2007; Schreiner et al., 2010). It was previously hypothesized that PAA might be produced from benzylglucosinolate via myrosinase and nitrilase activity (Ludwig-Müller and Cohen, 2002) but experimental data on *in planta* conversion of Bz-CN to PAA by nitrilases have to the best of our knowledge not yet been reported.

Here we describe the auxin-like effect of exogenously applied Bz-CN on Arabidopsis growth. Bz-CN treatment inhibited primary root growth, promoted adventitious root formation, and induced the expression of the DR5::GUS reporter and of auxin-responsive genes. Our results point to a nitrilase-catalyzed conversion of Bz-CN to PAA. Exogenously applied PAA phenocopied the auxin-like effects of Bz-CN, and nitrilase mutants of Arabidopsis showed reduced sensitivity to Bz-CN but not to PAA treatment. Mutant lines were used to test the involvement of known auxin response actors in the Bz-CN-triggered auxin response.

## Materials and Methods

### Chemicals

Benzyl cyanide (phenylacetonitrile; CAS 140-29-4; purity 98%; catalog number B19401), PAA (CAS 103-82-2; purity ≥ 98%; catalog number P6061), and other chemicals were purchased from Sigma-Aldrich unless stated otherwise.

### Plant Material and Growth Conditions

Mutant seeds for *NIT1* (*nit1-3*, N3738), *NIT2* (SAIL_681H09, N862966), and *NIT4* (SALK_016289, N516289) were obtained from the European Arabidopsis Stock Centre (NASC, Nottingham, United Kingdom). The EMS mutant *nit1-3* has been previously characterized (Normanly et al., 1997), and was verified by sequencing a PCR amplicon with the primers indicated in **Table 1**. The T-DNA insertion mutants for *NIT2* and *NIT4* were verified by PCR using T-DNA and gene specific primer combinations (**Table 1**). The *NIT2*RNAi knockdown line was described recently (Lehmann et al., 2017). The auxin reporter lines DR5::GUS (Ulmasov et al., 1997), DII-VENUS, and mDII-VENUS (Brunoud et al., 2012) have been described previously.

**Table 1:** Primers used for genotyping nitrilase mutants.

Mutant	Primer name	Primer sequence	Target
*nit1-3*	nit1-3_ko_FOR	TGCCGTTTGAAACATCTAGG	*NIT1*
	nit1-3_ko_REV	GAGTAATGTCCAACCGAATCG	*NIT1*
*nit2*	SAIL_681_H09_FOR	CTCCCGCCACTCTAGGTAATC	*NIT2*
	SAIL_681_H09_REV	AACCATCAGCAGTAGGTGCAC	*NIT2*
	LB3_SAIL	TAGCATCTGAATTTCATAACCAATCTCGATACAC	*SAIL T-DNA*
*nit4*	SALK016289_NIT4_F	TATGAAAGGCCCAGTGACTTG	*NIT4*
	SALK016289_NIT4_R	CACTTCAGGCCTCGAGTAATG	*NIT4*
	LBa1	TGGTTCACGTAGTGGGCCATCG	*SALK T-DNA*

The following auxin mutants were obtained from the European Arabidopsis Stock Centre (NASC, Nottingham, United Kingdom): *afb5-5* (N610643) (Prigge et al., 2016), *axr1-3* (N3075) and *axr1-12* (N3076) (Leyser et al., 1993), *tir1-1* (N3798) (Ruegger et al., 1998), *axr2-1* (N3077) (Nagpal et al., 2000), and *axr3-1* (N57504) (Leyser et al., 1996).

All lines were propagated in plant growth rooms under a 16 h light (75 μmol m^−2^ s^−1^) / 8 h dark photoperiod at 22/18°C.

### Treatments of Seedlings With Benzyl Cyanide and Phenylacetic Acid *in vitro*

Seeds were surface sterilized with chlorine gas (Clough and Bent, 1998) for 3 h and stratified for 3 days at 4°C in water. Seeds were subsequently sown on square plates (120 mm × 120 mm × 17 mm, Greiner Bio One) containing 75 mL solid half strength Murashige and Skoog medium [2.15 g L^−1^ MS basal salt mixture (Sigma-Aldrich, M5524); 20 g L^−1^ sucrose; pH 5.7; 10 g L^−1^ 2:1 mix of phyto agar (Duchefa Biochemie, P1003): bacteriological agar (VWR, 84609)]. Plates were sealed (3M Micropore Surgical Tape 1530-1) and placed in a vertical position into a controlled growth chamber (VB1514, Vötsch Industrietechnik) under a 16 h light (75 μmol m^−2^ s^−1^) / 8 h dark regime at 22/18°C for 10 days.

Seedlings were grown in parallel on half strength MS medium (control) and on half strength MS medium supplemented with either Bz-CN or PAA. For the evaluation of growth parameters and gene expression, several replicate plates (see text for specific details) were prepared for each of the treatments. Bz-CN and PAA were added to the final concentration(s) of 25, 50, and 100 μM (unless indicated otherwise in the text) to the medium immediately before pouring the plates. Seedling growth was monitored by taking pictures of the plates at the different time points indicated in the text and primary root length and hypocotyl length was measured using the ImageJ software (Schneider et al., 2012). In addition, total biomass of the seedlings was determined at day 10. The statistical difference between control and treatment groups was assessed by using SigmaPlot (version 13.0, Systat Software) software and for the details of the statistical tests see the corresponding figure legends.

### RNA Extraction and cDNA Synthesis

Root tissue (25–50 mg) from seedlings grown on control medium, medium supplemented with Bz-CN (25, 50, and 100 μM), or medium supplemented with PAA (25, 50, and 100 μM) was harvested on day 10 and flash frozen in liquid nitrogen. For each treatment, three replicate plates were harvested by pooling the root tissue from seedlings grown on the same plate. Frozen plant tissue was submitted to two disruption cycles with a TissueLyser II (Qiagen) for 2 min at 25 Hz. Total RNA was extracted with the Spectrum Plant Total RNA kit (Sigma-Aldrich) as described by the supplier, but with lysis solution being added to the plant tissue between the two disruption cycles. An on-column DNase digestion was performed using the RNase-Free DNase Set (Qiagen) to eliminate genomic DNA. Total RNA was quantified with a NanoDrop ND-1000. RNA was stored at −80°C until further processing.

cDNA synthesis was performed on 1 μg total RNA using the QuantiTect Reverse Transcription Kit (Qiagen) following the supplier’s instructions. After cDNA synthesis, reactions were diluted five times in ddH_2_O before being used in quantitative real-time PCR.

### Quantitative Real-Time PCR

Quantitative real-time PCR (qPCR) was performed on the three replicate cDNAs for each treatment on a LightCycler 480 using the LightCycler 480 SYBR Green I Master kit (Roche Applied Science). PCR parameters were as follows: pre-incubation at 95°C for 5 min, 45 amplification cycles with a 10 s incubation at 95°C, a 10 s incubation at 58°C, and a 10 s incubation at 72°C. Primers used to amplify target genes are given in **Table 2**. *TIP41-like* (*At4g34270*), *PP2A subunit A3* (*At1g13320*), and *Actin 2* (*At3g18780*) were used as reference genes (Czechowski et al., 2005) and their stability was assessed using geNorm implemented in the qbase+ (Biogazelle) software (Vandesompele et al., 2002). Cq values for each amplification curve and PCR efficiencies for each pair of primers were calculated using the LinRegPCR software version 2012.3 (Ramakers et al., 2003; Ruijter et al., 2009). The statistical significance of differences in gene expression levels between treated and control seedlings was determined by the qbase+ software version 2.6 (Hellemans et al., 2007). To verify the identity of amplicons, qPCR reactions were run on an agarose gel (1.5%) following standard procedures, amplicons were purified from the gel using the Wizard SV Gel and PCR Clean-Up System (Promega), sequencing reactions were prepared with the BigDye Terminator v3.1 Cycle Sequencing kit (Applied Biosystems), and sequencing was performed at the DNA Sequencing Core Facility of the University Hospital North-Norway (Tromsø, Norway).

**Table 2:** Primers used for qPCR analysis.

Target gene	Primer name	Sequence
*NIT1* (*At3g44310*)	NIT1_q_FOR	GTACGACGACAAAGAACATGATTC
	NIT1_q_REV	CCAAGATCAATATCAGCTGTGAC
*NIT2* (*At3g44300*)	NIT2_q_FOR	TACGACGACAAAGAGCCTGAC
	NIT2_q_REV	CATCACCAAGATCAAGATCAGCT
*NIT3* (*At3g44320*)	NIT3_qP_FN	CCTACTGCTGATTATTCGTTGG
	NIT3_qP_RN	TACGCTTGCAGAACTGGTG
*NIT4* (*At5g22300*)	NIT4_q_FOR	AAGAGAGCCTAACACCGGAC
	NIT4_q_REV	GTGCTATGTCCCCAAGATCTAG
*IAA5* (*At1g15580*)	IAA5_qP_FOR	CAATTTTGATGATACGTTGAAGG
	IAA5_qP_REV	AGGAACATTTCCCAAGGAAC
*IAA12* (*At1g04550*)	IAA12_BDL_qP_FOR	TCGTTCAAGTCAAGTGGTAGG
	IAA12_BDL_qP_REV	AACTTTCTTCTCCCCGTCTC
*IAA19* (*At3g15540*)	IAA19_qP_FOR	TTGGGGGATGTTTCTAGAGTC
	IAA19_qP_REV	CGTCTACTCCTCTAGGCTGC
*IAA29* (*At4g32280*)	IAA29_qP_FOR	CCGAATATGAAGATTGCGAC
	IAA29_qP_REV	CAAAGATCTTCCATGTAACATCC
*LBD16* (*At2g42430*)	LBD16_qP_FOR	AACAACAGGTGGCTTTCTTG
	LBD16_qP_REV	GGTACTTTCCGAGCTGTGTC
*LBD29* (*At3g58190*)	LBD29_qP_FOR	CAACAACAGGTTGTGAATTTACA
	LBD29_qP_REV	TCTGATGTTGGTGAATCAGC
*TIP41-like* (*At4g34270*)	TIP41-like FOR	GTGAAAACTGTTGGAGAGAAGCAA
	TIP41-like REV	TCAACTGGATACCCTTTCGCA
*PP2AA3* (*At1g13320*)	PP2A FOR	GGAGAGTGACTTGGTTGAGCA
	PP2A REV	CATTCACCAGCTGAAAGTCG
*Actin2* (*At3g18780*)	Actin2_qPCR_FOR	GCCATCCAAGCTGTTCTCTC
	Actin2_qPCR_REV	CAGTAAGGTCACGTCCAGCA

### GUS Staining

The β-glucuronidase (GUS) staining was performed as previously described (Scarpella et al., 2004), with minor modifications. Briefly, 10-days-old DR5::GUS seedlings were incubated in 90% (v/v) pre-chilled acetone at −20°C for 1 h and washed twice for 5 min with 100 mM sodium phosphate buffer (pH 7.8). GUS reaction buffer (100 mM sodium phosphate buffer, pH 7.8; 10 mM EDTA; 1 mM potassium ferrocyanide; 1 mM potassium ferricyanide; 1% Triton X-100) supplemented with 1 mM X-GlcA cyclohexylammonium salt (Duchefa Biochemie, X1405) was added and the samples were vacuum infiltrated for 3 min. After an overnight incubation in the dark at 37°C, the staining solution was replaced with a mixture of ethanol and acetic acid (3:1) and samples were incubated at room temperature overnight. Samples were then transferred to 70 % (v/v) ethanol and kept at 4°C prior to imaging with a Zeiss Axio Zoom.V16 fluorescence stereo zoom microscope equipped with a Plan-Neofluar Z 1.0x/0.25 (FWD 56 mm) objective using the ZEN 2012 (blue edition, ZEISS) software.

### Confocal Laser Scanning Microscopy of Benzyl Cyanide-Treated Seedlings

Col-0 WT, DII-VENUS, and mDII-VENUS seeds were sown on normal growth medium (control) and grown for seven days under controlled conditions as described above. On day 7, approximately half of the seedlings were transferred either to fresh growth medium (control) or to medium supplemented with Bz-CN at different concentrations (for details, see the text). Plates were sealed and transferred back into the growth chamber. Treated and non-treated seedlings were subjected to confocal laser scanning microscopy (CLSM) analysis after 24 and 48 h. Prior to imaging, seedlings were rinsed in ddH_2_O then stained with the cell viability marker, propidium iodide (PI, Molecular Probes P3566), at 500 nM final concentration (50 μM stock solution in ddH_2_O) for 1 min. Subsequently seedlings were rinsed again in ddH_2_O then mounted onto microscope glass slides. The yellow fluorescent protein (YFP) variant, VENUS, was detected by a Leica (DMI 6000 CS Bino inverted microscope with Adaptive Focus Control) True Confocal Scanner (TCS) SP8 system. HCX IRAPO L 25x/0.95 water immersion objective (WD 2.4 mm) was used with 1 airy unit (AU) pinhole size. Samples were excited with the white light laser (WLL, 470–670 nm) at 514 nm and fluorescence emission was detected in the 524–540 nm range using Leica HyD hybrid detector. PI was excited at 561 nm and the fluorescence was recorded between 571 and 715 nm. Images were processed using the Leica Application Suite X (LAS X 2.0).

## Results

### Exogenous Application of Benzyl Cyanide Leads to Auxin-Like Effects on Arabidopsis Seedlings *in vitro*

**FIGURE 1:**
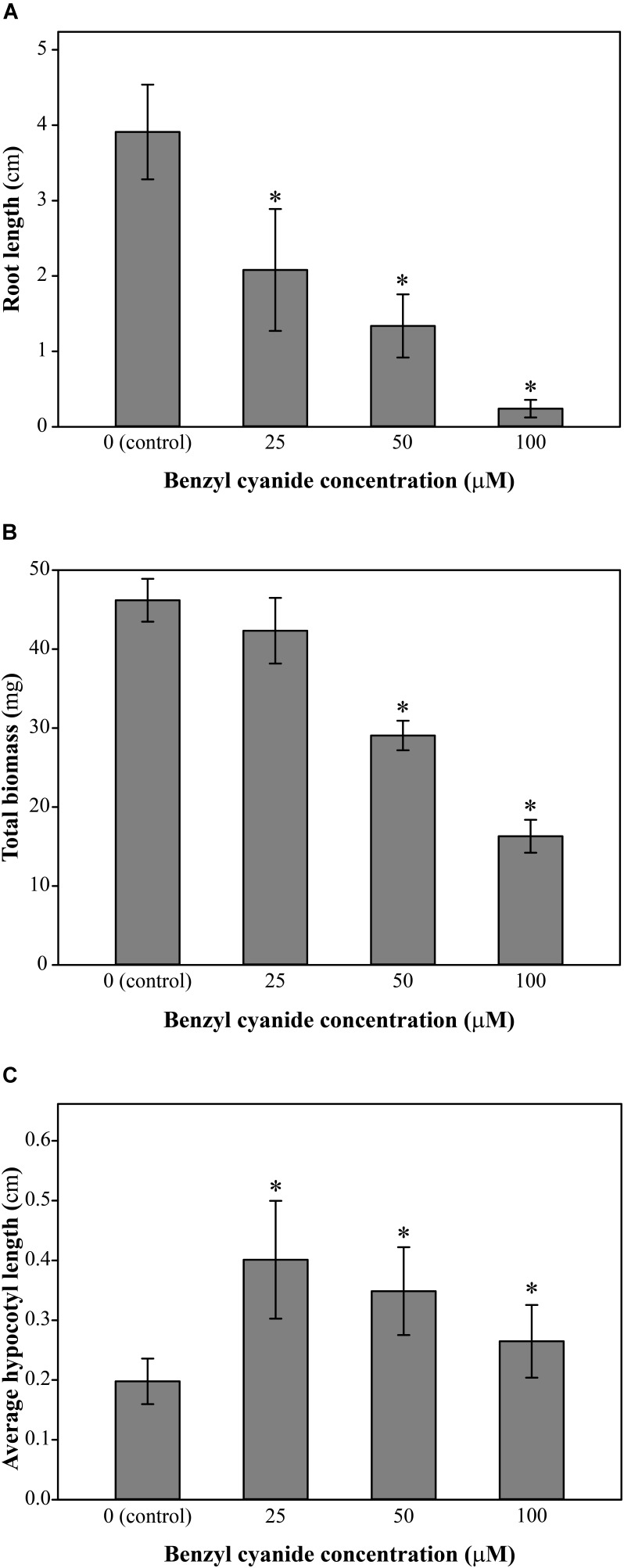
Effect of benzyl cyanide on Arabidopsis growth parameters. Col-0 seedlings were grown on solid *in vitro* medium supplemented with Bz-CN at the indicated concentrations and the length of the main root **(A)**, the total biomass **(B)**, and the length of the hypocotyl **(C)** were assessed on day 10. Values for root length are the average (±SD) of 60 seedlings (15 per replicate plate). Values for biomass are the average fresh weight (±SD) of 15 pooled seedlings from four replicate plates (*n* = 4). Values for hypocotyl length are the average (±SD) of 200 seedlings (50 per replicate plate). Stars indicate a statistically significant difference to the control treatment. (One-way ANOVA with a *post hoc* Holm–Sidak test; ^∗^*P* < 0.001. On the occasion when the sample groups did not pass the normality and/or equal variance test, Kruskal–Wallis one-way ANOVA on ranks with a *post hoc* Dunn’s test was used).

**FIGURE 2:**
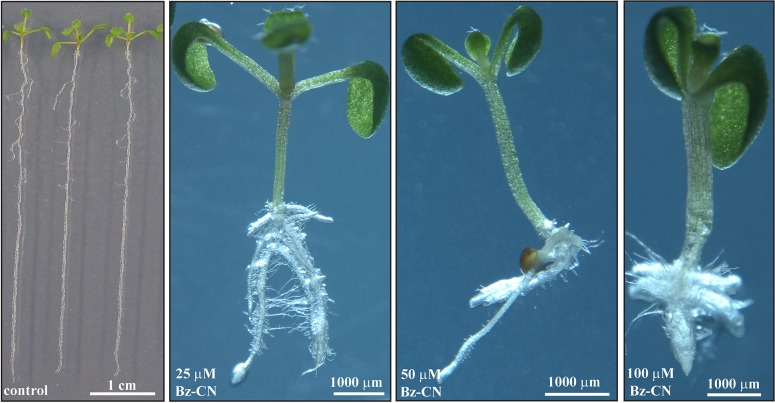
Benzyl cyanide treatment led to auxin-like effects in Arabidopsis. Pictures of representative Col-0 seedlings after 10 days on solid *in vitro* medium supplemented with Bz-CN at the indicated concentrations. Scale bars as shown.

Growing Arabidopsis Col-0 on solid *in vitro* medium supplemented with Bz-CN at concentrations ranging from 25 to 100 μM for 10 days led to a dose-dependent decrease in primary root length (**Figure 1A**) and total biomass (**Figure 1B**). Already at 25 μM Bz-CN, the primary root length was approximately half (53%) of that of non-treated seedlings, whereas at the two highest concentrations, these values were 34% and 6%, respectively. The reduction in total biomass was less severe (**Figure 1B**). A concentration of 25 μM Bz-CN led to a doubling of hypocotyl length (**Figure 1C**). The hypocotyl length of seedlings exposed to Bz-CN was longer at all doses tested but a gradual decrease could be observed toward elevating concentrations. In addition to a shortening of the primary root and longer hypocotyl, seedlings grown on Bz-CN presented epinastic cotyledons, had longer root hairs and developed numerous adventitious roots (**Figure 2** and **Supplementary Figure S1**). These phenotypes that were triggered by the exposure of Arabidopsis Col-0 seedlings to Bz-CN reminded of an auxin-like effect.

### Benzyl Cyanide Induces the Expression of Auxin-Responsive Genes

**FIGURE 3:**
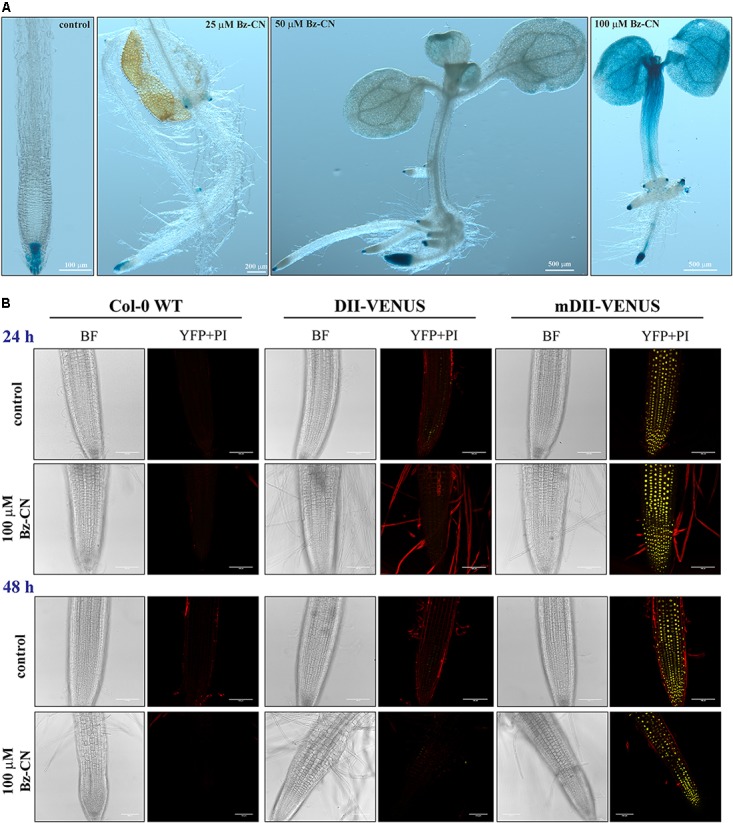
Response of auxin reporter lines when exposed to benzyl cyanide. Representative pictures of auxin reporter lines after GUS staining of DR5::GUS seedlings grown for 10 days on Bz-CN **(A)** and by fluorescence detection in DII-VENUS seedlings exposed to Bz-CN for 24 and 48 h **(B)**. Scale bars as indicated for images of GUS-stained seedlings, and 100 μm for CLSM pictures of roots.

In order to confirm that Bz-CN exposure leads to an auxin response, auxin reporter lines were grown for 10 days on medium supplemented with Bz-CN. As expected, auxin reporter lines did not differ in phenotype from the wild type under the same growth conditions (**Figures 2**, **3**). Roots of DR5::GUS seedlings grown on medium supplemented with Bz-CN showed an increase in GUS staining compared to the control treatment, indicating an elevated auxin response. GUS staining was also observed in root primordia originating from the hypocotyl at 50 and 100 μM Bz-CN (**Figure 3A**). At 100 μM Bz-CN, the GUS staining extended to the hypocotyl and the cotyledons. An auxin response upon exposure to Bz-CN was also confirmed by using the DII-VENUS reporter line. In this line, the fusion of the auxin-interaction domain DII to the YFP VENUS leads to the degradation of the fusion protein in response to auxin and thereby allows to visualize dynamic changes of auxin levels by relative fluorescence levels (Brunoud et al., 2012). In our assays, lower fluorescence was detected in DII-VENUS seedlings exposed to Bz-CN for 24 and 48 h than in seedlings grown on control medium. As expected, the fluorescence in the control line mDII-VENUS, where a mutated auxin insensitive variant of DII no longer leads to auxin-triggered degradation of VENUS, was not affected by the Bz-CN treatment (**Figure 3B**).

**FIGURE 4:**
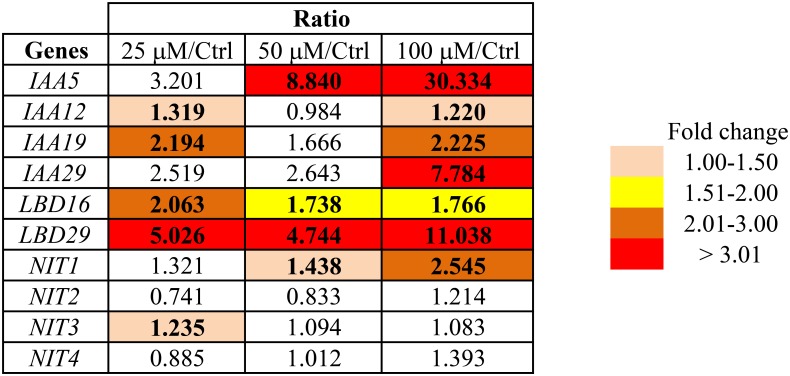
Transcriptional response of Arabidopsis to benzyl cyanide treatments. The expression of several auxin-induced genes and of the four genes encoding nitrilases was assessed by qPCR in seedlings grown for 10 days on Bz-CN-supplemented medium. Values represent fold changes of gene expression by Bz-CN treatment compared to control. Only statistically significant values (one-way ANOVA followed by Tukey’s *post hoc* test; *P* < 0.05) are highlighted.

In addition, we assessed the expression of several auxin-responsive genes in seedlings grown for 10 days on Bz-CN-supplemented medium by qPCR (**Figure 4**). The genes were selected based on their responsiveness to several auxin treatments (Paponov et al., 2008; Sugawara et al., 2015). Exposure to 50 and 100 μM Bz-CN induced the expression of *IAA5* (*INDOLE-3-ACETIC ACID INDUCIBLE 5; At1g15580*) by 8- and 30-fold, respectively, compared to the control treatment. *IAA19* (*At3g15540*) and *IAA29* (*At4g32280*) were also upregulated but to a lesser extent than *IAA5*, and *IAA12* (*At1g04550*) was hardly affected. The expression of the two auxin-responsive genes *LBD16* (*LATERAL ORGAN BOUNDARIES-DOMAIN 16*; *At2g42430*) and *LBD29* (*At3g58190*), encoding proteins involved in lateral root formation (Feng et al., 2012), was induced by all three Bz-CN concentrations (**Figure 4**).

### Exogenous Application of Phenylacetic Acid to Arabidopsis Phenocopies the Effects of Benzyl Cyanide

**FIGURE 5:**
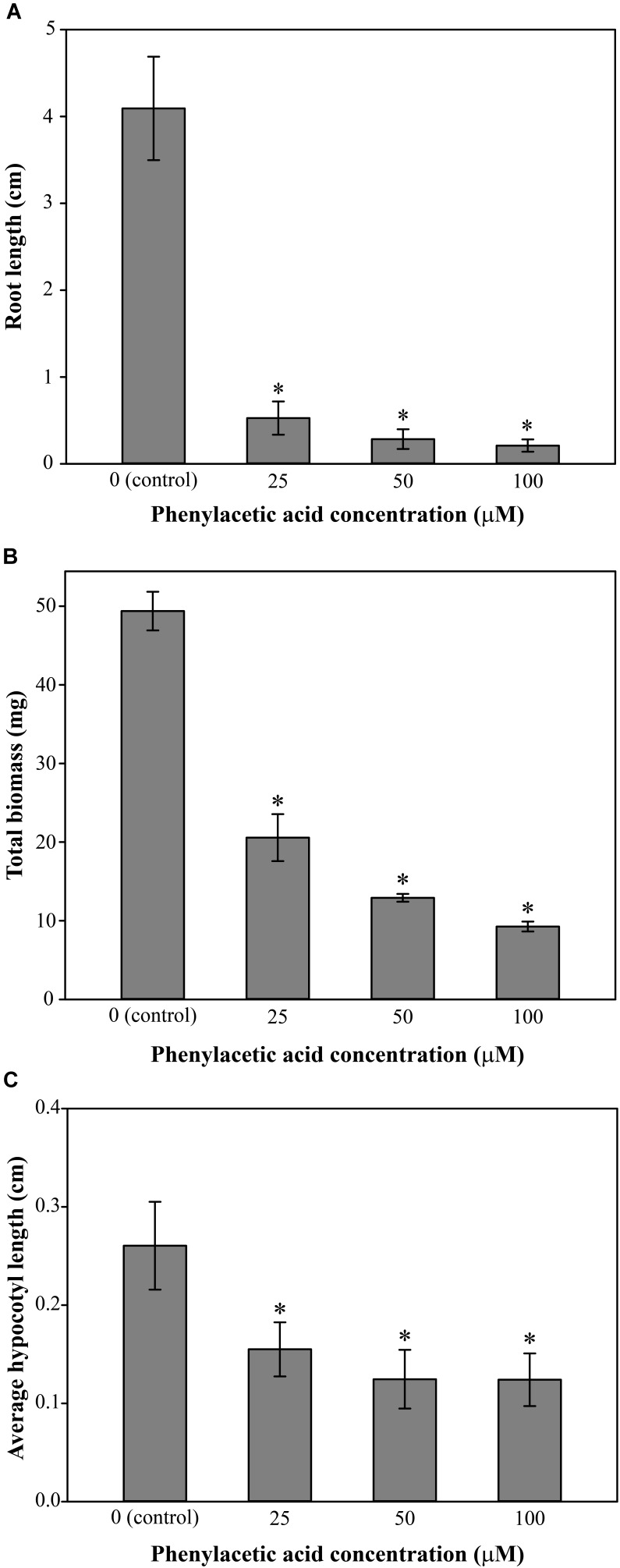
Effect of phenylacetic acid on Arabidopsis growth parameters. Col-0 seedlings were grown on solid *in vitro* medium supplemented with PAA at the indicated concentrations and the length of the main root **(A)**, the biomass **(B)**, and the length of the hypocotyl **(C)** were assessed on day 10. Values for root length are the average (±SD) of 60 seedlings (15 per replicate plate). Values for biomass are the average fresh weight (±SD) of 15 pooled seedlings from four replicate plates (*n* = 4). Values for hypocotyl length are the average (±SD) of 200 seedlings (50 per replicate plate). Stars indicate a statistically significant difference to the control treatment. (One-way ANOVA with a *post hoc* Holm–Sidak test; ^∗^*P* < 0.001. On the occasion when the sample groups did not pass the normality and/or equal variance test, Kruskal–Wallis one-way ANOVA on ranks with a *post hoc* Dunn’s test was run).

**FIGURE 6:**
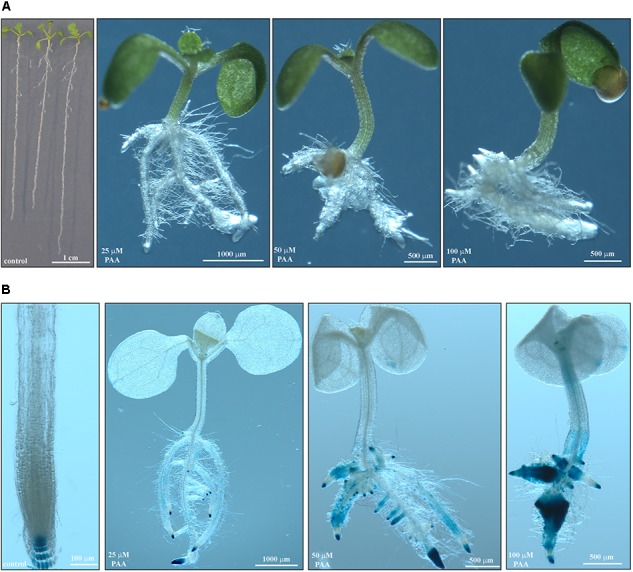
Auxin response in Arabidopsis triggered by phenylacetic acid. **(A)** Pictures of representative Col-0 seedlings after 10 days on solid *in vitro* medium supplemented with PAA at the indicated concentrations. **(B)** GUS-stained DR5::GUS seedlings grown for 10 days on medium containing various doses of PAA. Scale bars as shown.

As it was previously postulated that Bz-CN might be converted into the auxin PAA (Steiner, 1948; Ludwig-Müller and Cohen, 2002), morphology and growth parameters were assessed for Arabidopsis Col-0 when exposed to PAA. The effect on root length and biomass was more pronounced for PAA than for Bz-CN at equal doses (**Figures 1**, **5**). Primary root length was decreased by 87% already at 25 μM PAA compared to the control condition, and the inhibition on the two higher doses was even more severe (**Figure 5A**). Similar to what was observed for Bz-CN, the total biomass was less affected by the PAA treatment than the root length (**Figure 5B**). While Bz-CN promoted hypocotyl growth (**Figure 1C**), all tested PAA concentrations had an inhibitory effect (**Figure 5C**). Like Bz-CN, PAA led to epinastic cotyledons, longer root hairs, and the formation of adventitious roots (**Figure 6A**), again with a more pronounced effect for PAA than for Bz-CN at lower concentrations. In addition, PAA exposure triggered an auxin response at the transcriptional level which was confirmed by increased GUS staining in DR5::GUS seedlings (**Figure 6B**) and by qPCR analysis of auxin-responsive genes (**Supplementary Figure S2**).

### Mutants Impaired in Auxin Signaling Are More Resistant to Benzyl Cyanide Exposure

**FIGURE 7:**
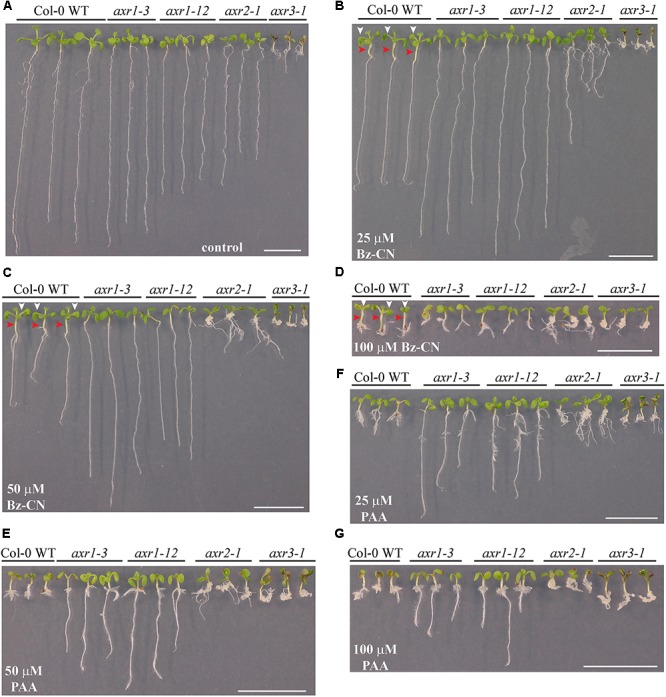
Response of mutants affected in auxin signaling to benzyl cyanide and phenylacetic acid exposure. Pictures of representative Col-0 wild type and auxin mutants after 10 days on solid *in vitro* control medium **(A)** and medium supplemented with Bz-CN **(B–D)** or PAA **(E–G)** at the indicated concentrations. In case of WT seedlings, red arrowheads mark the elongated hypocotyls, whereas the white ones point to the epinastic cotyledons. Scale bars denote 1 cm.

To further characterize the auxin response triggered by Bz-CN, mutants affected in auxin signaling were grown on Bz-CN-supplemented medium. Mutants affected in either TIR1 or AFB5, two of the six F-box proteins that participate in auxin signaling (Parry et al., 2009), did not differ in their response to Bz-CN from that of wild type seedlings (**Supplementary Figures S3, S4**). The two auxin resistant mutants *axr1-3* and *axr1-12* which are deficient in auxin signaling (Lincoln et al., 1990; Dharmasiri et al., 2007) were on the other hand less inhibited in their root growth than wild type when exposed to Bz-CN (**Figure 7**). At the highest Bz-CN concentration (i.e., 100 μM), no difference in root length was, however, observed between *axr1* mutant and wild type seedlings. While treatment with Bz-CN resulted in longer hypocotyls and epinastic cotyledons in wild type seedlings, these morphological features could not be observed in the *axr1* mutants (**Figures 7B–D**). Due to their inherent mutation, the two additional mutants *axr2-1* and *axr3-1* exhibited characteristic phenotypes already on the control medium (**Figure 7A**), but they were not resistant to Bz-CN. However, the two mutants had aberrant growth morphology especially at the hypocotyl and/or hypocotyl–root junction resembling an expansion/aggregation of cells in those regions (**Figures 7B–D**).

When the *axr* mutants were exposed to PAA, the exhibited phenotype was more severe than seen for Bz-CN treatment (**Figures 7E–G**). The *axr1-3* and *axr1-12* lines had longer roots at all doses tested compared to the wild type. The strange growth features observed for *axr2-1* and *axr3-1* on Bz-CN were even more visible on PAA.

### Nitrilase Mutants Show Insensitivity to Benzyl Cyanide but Not to Phenylacetic Acid

**FIGURE 8:**
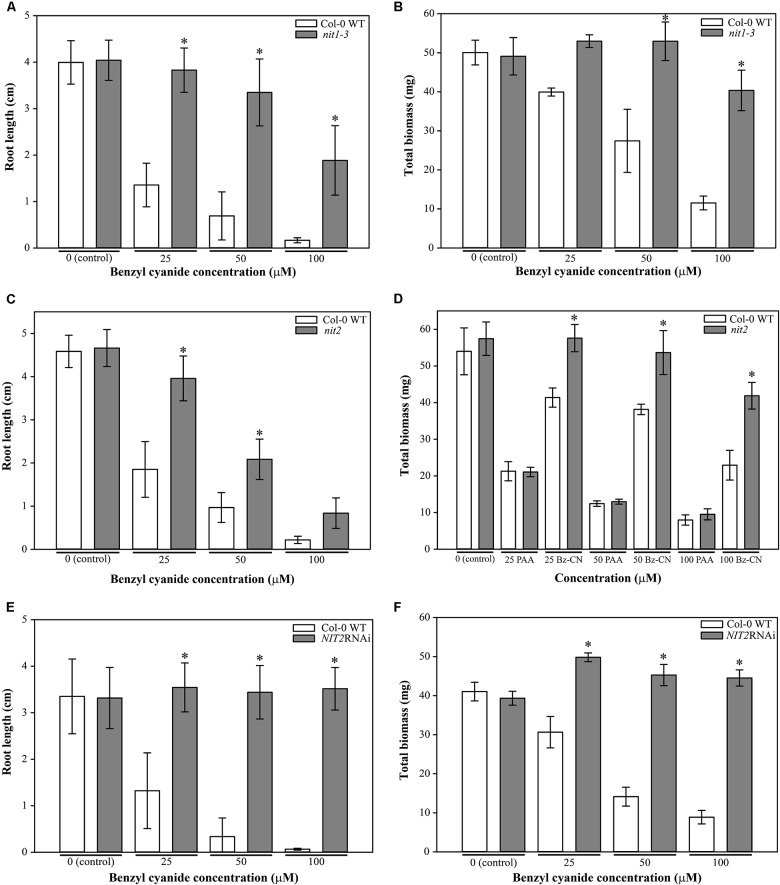
Effect of benzyl cyanide or phenylacetic acid on root length and biomass of the *nit1-3* and *nit2* mutants, and the *NIT2*RNAi knockdown line. Col-0, *nit1-3*, *nit2*, and *NIT2*RNAi seedlings were grown on solid *in vitro* medium supplemented with Bz-CN or PAA at the indicated concentrations and the length of the main root **(A,C,E)** and the total biomass **(B,D,F)** were assessed on day 10. Values for root length are the average (±SD) of 60 seedlings (15 per replicate plate). Values for biomass are the average fresh weight (±SD) of 15 pooled seedlings from four replicate plates (*n* = 4). Stars indicate a statistically significant difference to the wild type. (One-way ANOVA with a *post hoc* Holm–Sidak test; ^∗^*P* < 0.001. On the occasion when the sample groups did not pass the normality and/or equal variance test, Kruskal–Wallis one-way ANOVA on ranks with a *post hoc* Dunn’s test was used).

**FIGURE 9:**
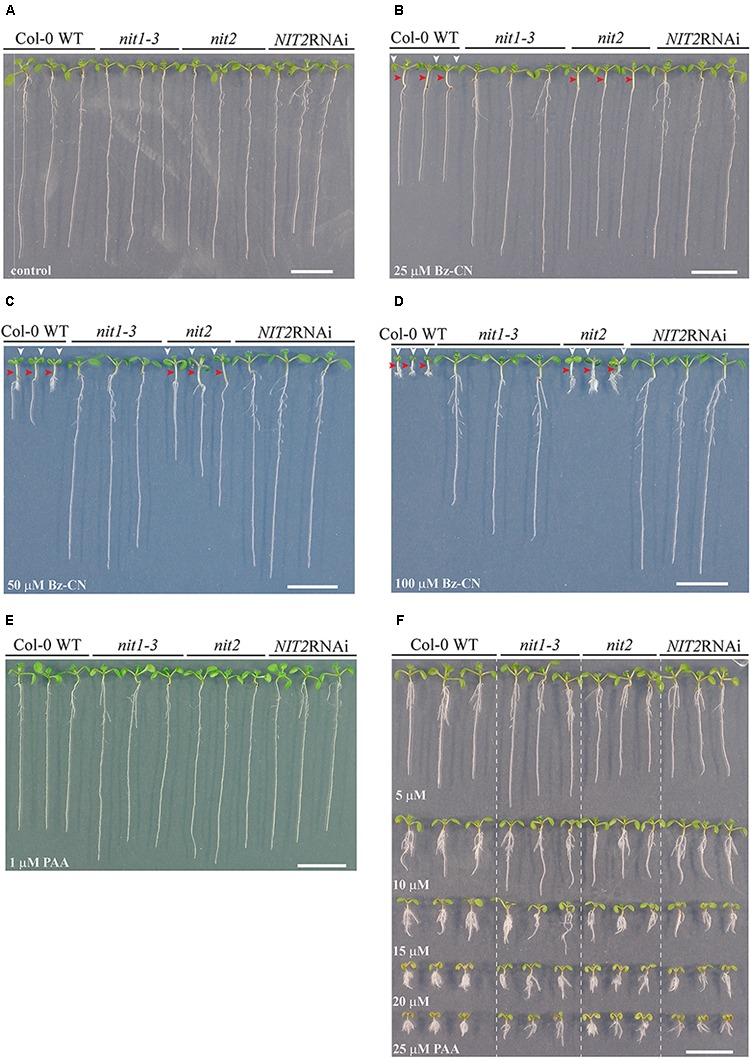
Effect of knocking out nitrilases on Arabidopsis growth when exposed to benzyl cyanide or phenylacetic acid. Pictures of representative wild type, *nit1-3*, *nit2*, and *NIT2*RNAi seedlings after 10 days grown on control medium **(A)** or on medium supplemented with Bz-CN **(B–D)** or PAA **(E,F)** at the given doses. In case of Bz-CN-treated WT and *nit2* seedlings, red arrowheads denote elongated hypocotyls, whereas the white ones mark epinastic cotyledons. Scale bars indicate 1 cm.

Next, we wanted to test whether the auxin-like effects seen after Bz-CN treatment were due to the conversion of Bz-CN to PAA *in planta* by Arabidopsis nitrilases. Therefore, the effects of Bz-CN and PAA were assessed on mutants affected in the expression of Arabidopsis nitrilases. Mutations in *NIT1* and *NIT2* and reduced nitrilase expression in the *NIT2*RNAi line inhibited the Bz-CN-triggered phenotypes to a large extent but these three lines differed in their response to Bz-CN (**Figures 8**, **9**).

The *nit1-3* mutant seedlings showed significantly longer roots and higher biomass on Bz-CN than wild type seedlings (**Figures 8A,B**). In addition, the *nit1-3* mutant showed less adventitious root formation than the wild type (**Figures 9A–D**). The hypocotyl of *nit1-3* mutant seedlings was not elongated and the cotyledons did not exhibit the characteristic epinastic growth following Bz-CN treatment (**Figures 9A–D**). While the *nit2* mutant also showed a reduced sensitivity to Bz-CN-triggered root inhibition compared to wild type (**Figure 8C**), the effect was less pronounced than for *nit1-3*. The *nit2* mutant had also significantly higher biomass than wild type under Bz-CN treatment (**Figure 8D**). Interestingly, in opposition to what was observed for *nit1-3*, the hypocotyls of *nit2* were elongated under Bz-CN treatment (**Figures 9A–D**). The *NIT2*RNAi knockdown line capable of suppressing several nitrilases (Lehmann et al., 2017) showed the highest insensitivity to Bz-CN as its root growth and biomass production was not inhibited by the tested concentrations (**Figures 8E,F**, **9**). In contrast, the effects of Bz-CN on the *nit4* mutant were similar to those on wild type (**Supplementary Figure S5**).

To see if exposure to Bz-CN affects the expression of the four nitrilase genes, qPCR analysis was performed on Col-0 seedlings. Only *NIT1* showed a clear induction by Bz-CN, with a maximum of 2.5-fold on 100 μM (**Figure 4**). In contrast, PAA led to a higher expression of *NIT4* but only slightly induced *NIT1* at the 100 μM concentration, and even repressed the expression of *NIT3* (**Supplementary Figure S2**).

In addition to the effect of Bz-CN, we tested the effect of PAA on nitrilase mutants (**Figures 9E,F**). All showed a strong auxin-like phenotype with epinastic cotyledons, a short primary root and adventitious root formation already at 10 μM PAA. There was no clear macroscopic distinction in the phenotype observed for wild type and any of the nitrilase mutants on the tested PAA concentrations (**Figure 9**). The *nit2* mutant was chosen as representative line to quantitatively assess the effect of PAA on Bz-CN insensitive nitrilase mutants. PAA affected the total biomass of *nit2* to a similar extent than that of wild type seedlings (**Figure 8D**).

## Discussion

We have shown here that Bz-CN and PAA exerted auxin-like effects on Arabidopsis Col-0 seedlings by affecting primary root length, total biomass, hypocotyl length, and adventitious root formation (**Figures 1**, **2**). PAA inhibited root elongation at 25 μM (**Figures 5A**, **6A**), which is consistent with earlier reports on Arabidopsis (Sánchez-Parra et al., 2014; Sugawara et al., 2015). Bz-CN also led to reduced primary root length but the response was less severe than for PAA at equal concentrations (**Figures 1**, **5**). Also, while all tested PAA concentrations had an inhibitory effect on Arabidopsis hypocotyls, the same concentrations of Bz-CN promoted hypocotyl elongation. Interestingly, Bz-CN was previously reported to be less toxic than PAA at higher doses and more effective than PAA at lower doses in promoting the elongation of *L. sativum* hypocotyl sections and adventitious root formation (Wheeler, 1977).

The auxin reporter lines DR5::GUS and DII-VENUS (**Figure 3**) as well as qPCR analysis of the expression of auxin-responsive genes (**Figure 4**) confirmed that the Bz-CN treatment triggered an auxin response. The genes assessed by qPCR showed significantly higher expression under at least one of the Bz-CN concentrations (**Figure 4**). Among these genes, the expressions of *IAA5* and *LBD29* were most highly induced by Bz-CN, which is consistent with previous reports on gene expression changes under different IAA treatments (Paponov et al., 2008). As expected, PAA also induced GUS expression in the DR5::GUS marker line (**Figure 6B**) and affected the expression of auxin-responsive genes such as *IAA5* and *LBD29* (**Supplementary Figure S2**), corroborating results from an earlier report (Sugawara et al., 2015).

As recombinant Arabidopsis nitrilases are capable of using Bz-CN as substrate *in vitro* (Vorwerk et al., 2001), we hypothesized that *in planta* conversion of Bz-CN to PAA by nitrilases was responsible for its auxin-like effect. The *nit1-3* and *nit2* mutants showed a reduced sensitivity to the Bz-CN treatment but not to PAA in comparison with wild type and *nit4*, which provides genetic evidence that nitrilases of the NIT1-NIT3 subgroup restricted to Brassicaceae are able to catalyze this reaction *in vivo*. This situation is similar to a reduced sensitivity that the *nit1-3* mutant exhibits toward IAN but not toward IAA (Normanly et al., 1997). Also, tobacco plants expressing Arabidopsis NIT2 have previously been shown to be able to convert exogenously supplied IAN to IAA and to exhibit an auxin-overproducing phenotype when grown on IAN (Schmidt et al., 1996; Dohmoto et al., 2000). The two nitrilase mutants, *nit1-3* and *nit2*, showed interesting quantitative and qualitative differences in their response to Bz-CN (**Figures 8**, **9**). This might be due to different *in vivo* preferences of the nitrilases for Bz-CN, like *in vitro* where NIT2 showed a slightly higher nitrilase activity than NIT1 on Bz-CN (Vorwerk et al., 2001). Differences in protein levels, intracellular localisations, or expression pattern between nitrilases (Bartel and Fink, 1994; Bartling et al., 1994; Kutz et al., 2002) might also have contributed to these phenotypic differences. The strong Bz-CN resistant phenotype of *nit1-3* in addition to the fact that *NIT1* expression was induced by the Bz-CN treatment, while none of the other nitrilases responded to the treatment, suggests that NIT1 is the major nitrilase converting Bz-CN to PAA in Arabidopsis.

As single *nit1-3* and *nit2* mutants were clearly distinguishable from wild type plants, despite overlapping expression patterns of *NIT1* and *NIT2* in Arabidopsis (Bartel and Fink, 1994) and similar substrate preferences for the recombinant nitrilases *in vitro* (Vorwerk et al., 2001), these nitrilases do not seem to be simply redundant *in vivo*. Each of these single nitrilase mutants is, to some extent, insensitive to Bz-CN, which might indicate that NIT heterocomplexes are required for optimal nitrilase activity in Arabidopsis. *Brassica napus* and *B. rapa* ssp. *pekinensis* nitrilases exist as multimers (Bestwick et al., 1993; Grsic et al., 1999), recombinant Arabidopsis NIT1 forms homomeric complexes *in vitro* (Osswald et al., 2002), and Arabidopsis NIT1 has been found in complexes with NIT2 *in planta* (Doskocilova et al., 2013). NIT4 homologs in Poaceae are known to form heterocomplexes to exert β-cyano-L-alanine hydrolyzing activity (Jenrich et al., 2007; Janowitz et al., 2009) and some fungal and bacterial nitrilases have been reported to form complexes (O’Reilly and Turner, 2003). It should, however, be noted that individual recombinant Arabidopsis nitrilases show activity *in vitro* (Bartling et al., 1992; Vorwerk et al., 2001; Osswald et al., 2002). To what extent the (sub)cellular localization of different nitrilases allows for heterocomplex formation *in planta* also merits further investigation.

The mutant affected in the *NIT4* nitrilase gene of Arabidopsis showed a similar response towards Bz-CN than the wild type (**Supplementary Figure S5**). This was expected as NIT4 has a strong substrate specificity for β-cyano-L-alanine (Piotrowski et al., 2001), an intermediate in the detoxification of cyanide produced during ethylene biosynthesis. NIT4 does not convert IAN to IAA *in vitro* (Vorwerk et al., 2001) and is therefore unlikely to play a major role in the conversion of Bz-CN to PAA *in vivo*. This also indicates that the *in planta* conversion of Bz-CN to PAA leading to the phenotypic effects seen in our assays is of an enzymatic nature and mediated by nitrilases of the NIT1-NIT3 subgroup. The fact that the Arabidopsis mutant affected in NIT4 did not exhibit reduced sensitivity to Bz-CN is also consistent with the absence or delay of auxin effects triggered by Bz-CN on sugar beet hypocotyl and pea epicotyl sections (Wheeler, 1977). As *nit1-3* and *nit2* were less sensitive than wild type to Bz-CN but indistinguishable on PAA, a non-enzymatic conversion of Bz-CN to PAA prior to uptake by the plant did not contribute substantially to the auxin-like effect of Bz-CN. This is further strengthened by the fact that the *NIT2*RNAi seedlings knocked down in functional nitrilases of the NIT1-NIT3 subgroup were not affected at all upon Bz-CN treatment, but they were indistinguishable from that of single mutants and wild type when exposed to PAA (**Figures 8**, **9**).

We postulate that the auxin effects seen in our experiments were due to a direct effect of free PAA but cannot exclude an indirect effect such as PAA affecting IAA synthesis or transport (Johnson and Morris, 1987; Morris and Johnson, 1987). Degradation products of PAA or conjugated PAA that might be formed inside the plant could also contribute to the effect (Morris and Johnson, 1987). PAA conjugates have been detected in *T. majus* and Arabidopsis, although the full range of PAA conjugates is not yet known (Jentschel et al., 2007; Sugawara et al., 2015; Staswick et al., 2017). Members of the GH3 auxin-amino acid conjugate synthases of rice and Arabidopsis have been reported to use PAA as a substrate *in vitro* (Staswick et al., 2005; Chen et al., 2010; Westfall et al., 2016). Several GH3-encoding genes were highly upregulated upon PAA treatment of Arabidopsis (Sugawara et al., 2015) and modifying the levels of certain *GH3*s (by overexpression or knockout mutants) affects the levels of free and conjugated PAA (Sugawara et al., 2015; Westfall et al., 2016; Staswick et al., 2017). Differentially localized processing of PAA in various seedling parts, as was described for IAA (Ludwig-Müller, 2011; Pencik et al., 2013; Zheng et al., 2016), might also explain why Bz-CN leads to different effects in roots and shoots of the *nit1-3* and *nit2* mutants (**Figure 9**).

To further characterize the Bz-CN-triggered auxin response, the behavior of some auxin mutants was tested upon exposure to Bz-CN (**Figure 7**). IAA is perceived by the SCF^TIR1/AFBs^-Aux/IAA (SKP1-CULLIN1-F-BOX, TRANSPORT INHIBITOR RESPONSE 1/AUXIN SIGNALING F-BOX-AUXIN /INDOLE-3-ACETIC ACID INDUCIBLE) co-receptors. Binding of IAA to TIR1/AFBs, which are part of the SCF E3 ligase complex, increases their interaction with members of the Aux/IAA family leading to the polyubiquitination and subsequent degradation of the latter proteins (for review: Salehin et al., 2015).

The mutants *axr1-3* and *axr1-12*, which are resistant to auxin as they are affected in RUB (RELATED TO UBIQUITIN)-mediated modification of CUL1 (CULLIN 1; Dharmasiri et al., 2007), were also more resistant to Bz-CN (**Figure 7**), indicating that PAA has a similar effect than other natural and synthetic auxins on *axr1* mutants and acts through similar mechanisms of auxin signaling. PAA has been shown to act through the TIR/AFB auxin-perception pathway (Sugawara et al., 2015), but we did not observe a difference in the growth response of the two auxin signaling mutants *tir1-1* and *afb5-5* to Bz-CN (**Supplementary Figures S3, S4**). As the TIR/AFB family consists of six members (Dharmasiri et al., 2005; Calderón Villalobos et al., 2012) higher order mutants might be necessary in order to see a difference in response to Bz-CN (Parry et al., 2009; Chapman et al., 2012; Sugawara et al., 2015). On the other hand, *tir1-1* and *afb5-5* single mutants have been shown to be more resistant than wild type plants, respectively, to the synthetic auxins 2,4-dichlorophenoxyacetic acid and picloram (Walsh et al., 2006; Parry et al., 2009; Prigge et al., 2016). In contrast to the *axr1* lines, the a*xr2-1* and *axr3-1* mutants – deficient, respectively, in IAA7 and IAA17 which act as repressors of auxin-inducible gene expression (Leyser et al., 1996; Nagpal et al., 2000) – were neither resistant to Bz-CN nor to PAA (**Figure 7**). PAA had stronger effects on these mutants at lower doses than Bz-CN, but treatment with the latter compound did not induce the characteristic growth features such as hypocotyl elongation or epinastic cotyledons as observed for the wild type and *nit2*. Instead, aberrant cell expansion was noticed in certain regions of the seedlings that might be explained by the mutation itself exacerbated by additional alteration in auxin homeostasis due to the nitrilase-mediated conversion of Bz-CN to PAA. While the *axr1* lines are interrupted in the perception of IAA – and presumably PAA too – at an early step of auxin signaling, the defects in *AXR2* or *AXR3* are located downstream of auxin perception; thus, it might serve as potential explanation for the enhanced resistance of *axr1* mutants toward Bz-CN/PAA but it needs to be further investigated.

## Conclusion

**FIGURE 10:**
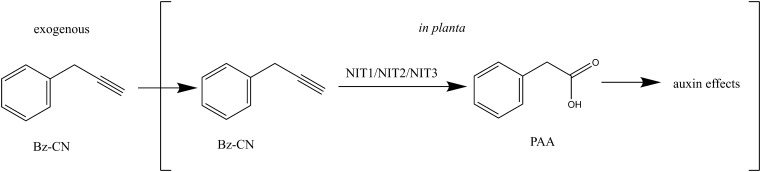
Model on how exogenous application of Bz-CN leads to auxin-like effects.

We have shown here that exogenous application of Bz-CN leads to auxin-like effects in Arabidopsis. Genetic evidence indicates that these effects are due to Bz-CN conversion into PAA *in planta* by nitrilase(s) of the NIT1-NIT3 subgroup restricted to Brassicaceae (**Figure 10**). PAA, a natural auxin, then triggers a response that includes the induction of auxin-responsive genes and morphological changes that are characteristic of auxin overexpression mutants, such as reduced primary root growth, lateral root formation, hypocotyl elongation, and epinastic cotyledons. While recent evidence argues against nitrilases playing a major role in auxin biosynthesis, it remains to be determined whether Bz-CN is an intermediate in the synthesis of physiologically relevant PAA levels in benzylglucosinolate-producing plants. The Bz-CN/nitrilase system can thus be helpful in investigating the mechanisms underlying PAA effects in plants.

## Author Contributions

JU and RK conceived and designed the research and analyzed the data. JU carried out the experiments. RK drafted the manuscript. JU and AB revised the manuscript. All authors read and approved the manuscript.

## Conflict of Interest Statement

The authors declare that the research was conducted in the absence of any commercial or financial relationships that could be construed as a potential conflict of interest.
