# Supplementary material for: Benzyl Cyanide Leads to Auxin-Like Effects Through the Action of Nitrilases in Arabidopsis thaliana
Source: Front Plant Sci. 2018 Aug 24;9:1240. doi: 10.3389/fpls.2018.01240 (PMC6117430; doi:10.3389/fpls.2018.01240)
Supplement: Supplementary file 1 [file Data_Sheet_1.pdf]

Supplementary Material

Benzyl Cyanide Leads to Auxin-like Effects Through the Action of Nitrilases in *Arabidopsis thaliana*

János Urbancsok, Atle M. Bones, Ralph Kissen*

Correspondence: Ralph Kissen: ralph.kissen@ntnu.no; ralph.kissen@bio.ntnu.no

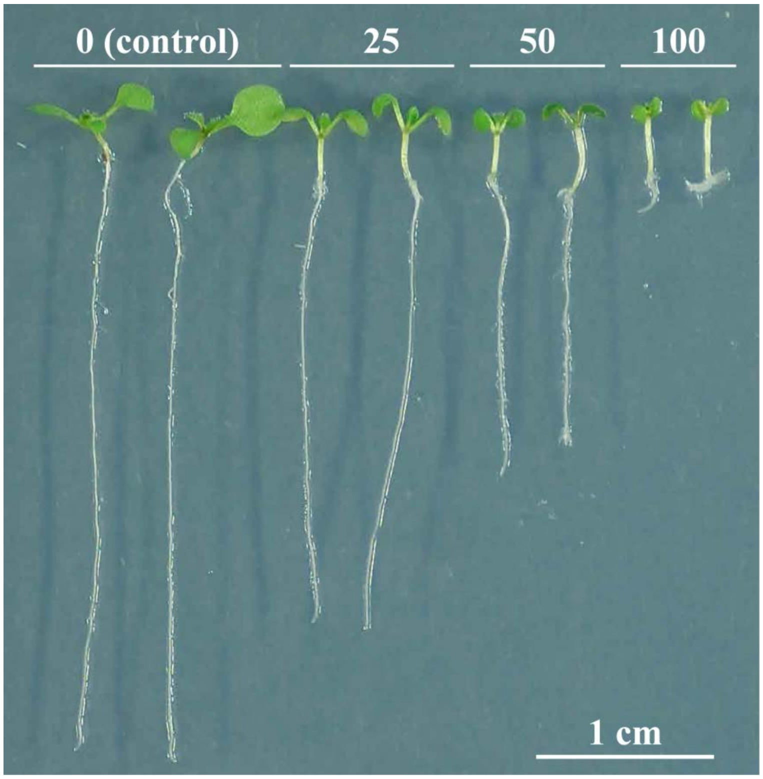

Supplementary Figure S1. Overview picture of Col-0 seedlings after 10 days on solid *in vitro* medium supplemented with Bz-CN at the indicated µM concentrations.

	Ratio		
**Genes**	25 µM/Ctrl	50 µM/Ctrl	100 µM/Ctrl
IAA5	**2.290**	**3.167**	**1.835**
IAA12	1.171	1.080	1.196
IAA19	**3.554**	**5.429**	**5.247**
IAA29	**5.151**	**10.889**	**14.327**
LBD16	**1.889**	**1.373**	**1.964**
LBD29	**8.743**	**7.181**	**4.235**
NIT1	1.153	1.370	**1.720**
NIT2	0.870	1.165	1.520
NIT3	0.890	**0.761**	**0.624**
NIT4	1.643	**2.049**	**2.484**

Fold change
0.50-1.00
1.01-1.50
1.51-2.00
2.01-3.00
>3.01

Supplementary Figure S2. Transcriptional response of *Arabidopsis thaliana* Col-0 to PAA treatments. The expression of several auxin-induced genes and of the four genes encoding nitrilases was assessed by qPCR in seedlings grown for 10 days on PAA-supplemented medium. Values represent fold changes of gene expression by PAA treatment compared to control. Statistically significant values ($P<0.05$; one-way ANOVA followed by Tukey's *post hoc* test) are highlighted.

A

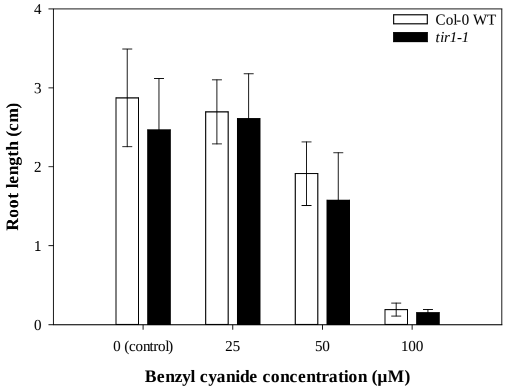

B

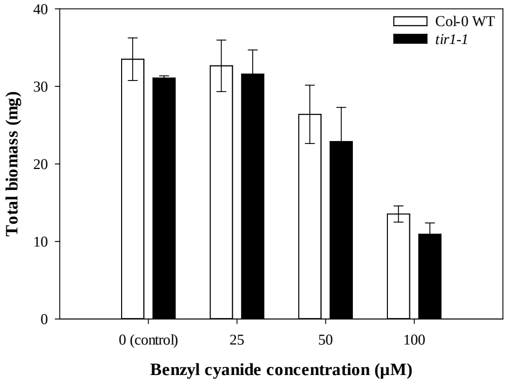

C

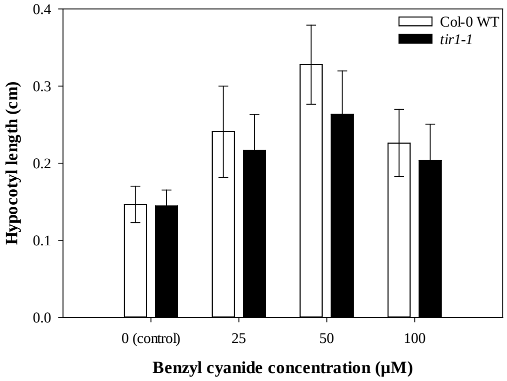

Supplementary Figure S3. Effect of Bz-CN on the *tir1-1* mutant. Col-0 and *tir1-1* seedlings were grown on solid *in vitro* medium supplemented with Bz-CN at the indicated concentrations and the length of the main root (A), the total biomass (B) and the length of the hypocotyl (C) were assessed on day 10. Values for root length and hypocotyl length are the average (± SD) of seedlings from four replicate plates (*n*= 48). Values for biomass are the average fresh weight (± SD) of 15 pooled seedlings from four replicate plates (*n*= 4). No statistically significant differences between the *tir1-1* mutant and the Col-0 wild type for a given treatment were detected ($P<0.001$; one-way ANOVA with a *post hoc* Holm-Sidak test or Kruskal-Wallis one-way ANOVA on Ranks with a *post hoc* Dunn's test when the sample groups did not pass the normality and/or equal variance test).

A

B

C

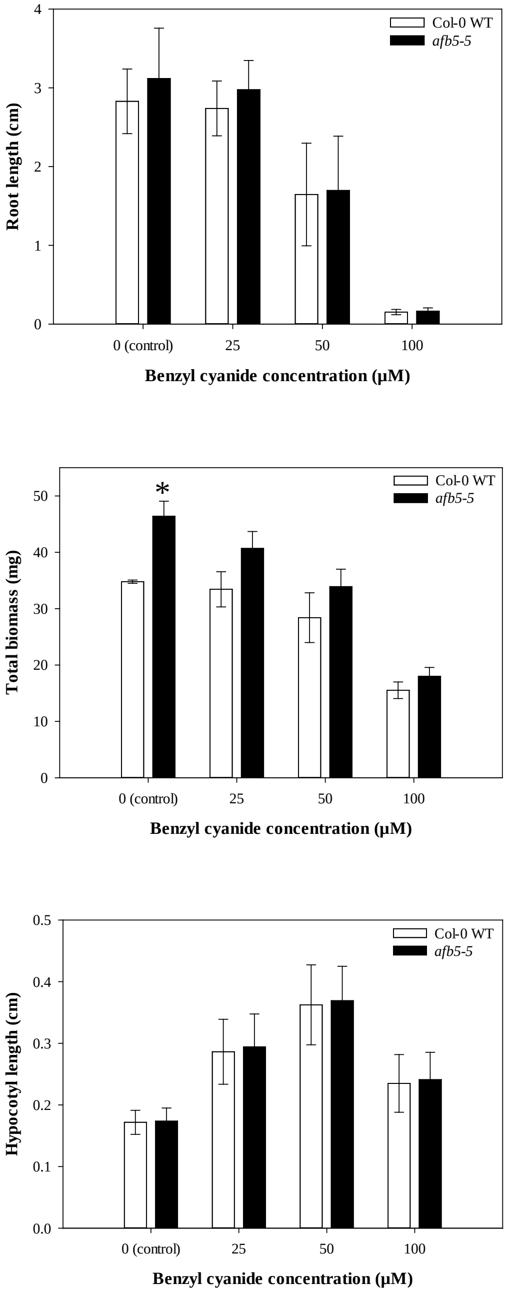

Supplementary Figure S4. Effect of Bz-CN on the *afb5-5* mutant. Col-0 and *afb5-5* seedlings were grown on solid *in vitro* medium supplemented with Bz-CN at the indicated concentrations and the length of the main root (A), the total biomass (B) and the length of the hypocotyl (C) were assessed on day 10. Values for root length and hypocotyl length are the average (± SD) of seedlings from four replicate plates (*n*= 48). Values for biomass are the average fresh weight (± SD) of 15 pooled seedlings from four replicate plates (*n*= 4). A star indicates a statistically significant difference between the *afb5-5* mutant and the Col-0 wild type for the given treatment (* P<0.001; one-way ANOVA with a *post hoc* Holm-Sidak test or Kruskal-Wallis one-way ANOVA on Ranks with a *post hoc* Dunn's test when the sample groups did not pass the normality and/or equal variance test).

A

B

C

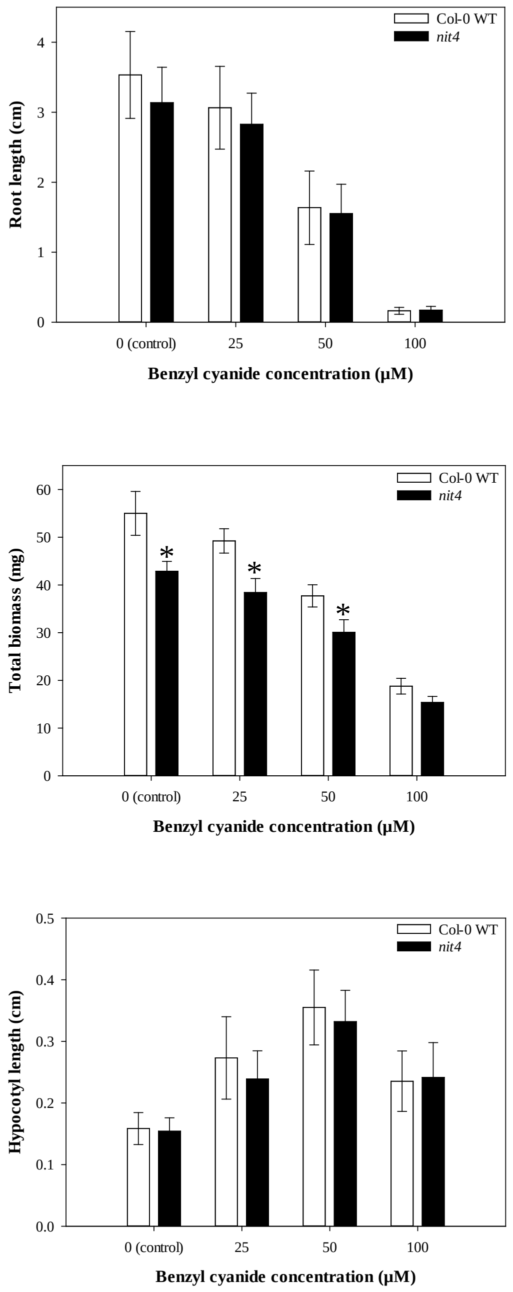

Supplementary Figure S5. Effect of Bz-CN on the *nit4* mutant. Col-0 and *nit4* seedlings were grown on solid *in vitro* medium supplemented with Bz-CN at the indicated concentrations and the length of the main root (A), the total biomass (B) and the length of the hypocotyl (C) were assessed on day 10. Values for root length and hypocotyl length are the average (± SD) of seedlings from four replicate plates (*n*= 48). Values for biomass are the average fresh weight (± SD) of 15 pooled seedlings from four replicate plates (*n*= 4). Stars indicate a statistically significant difference between the *nit4* mutant and the Col-0 wild type for the given treatment (* P<0.001; one-way ANOVA with a *post hoc* Holm-Sidak test or Kruskal-Wallis one-way ANOVA on Ranks with a *post hoc* Dunn's test when the sample groups did not pass the normality and/or equal variance test).
